# MR imaging of subaortic and retroesophageal anomalous courses of the left brachiocephalic vein in the fetus

**DOI:** 10.1038/s41598-018-33033-6

**Published:** 2018-10-03

**Authors:** Su-Zhen Dong, Ming Zhu

**Affiliations:** Department of Radiology, Shanghai Children’s Medical Center, Shanghai Jiaotong University School of Medicine, Shanghai, 200127 China

## Abstract

The purpose of this study was to report fetal cases of subaortic and retroesophageal anomalous courses of the left brachiocephalic vein (LBCV) evaluated by fetal cardiac magnetic resonance imaging (MRI). A retrospective review of 7282 fetal cardiac MRI from June 2006 to March 2017, nine cases of anomalous courses of the LBCV were correctly diagnosed by fetal cardiac MRI, one case of abnormal subaortic left brachiocephalic vein (ASLBV) missed by fetal MRI was identified postnatally during further imaging of the TOF. The diagnosis was confirmed postnatally by cardiac CT/MRI. An ASLBV was found in 8 cases, a retroesophageal LBCV was found in 2 additional cases with right aortic arch and aberrant left subclavian artery. 3 of 8 ASLBV cases were with a right aortic arch, 4 ASLBV cases had additional cardiovascular anomalies with one case isolated. 7 of 8 ASLBV and 2 retroesophageal LBCV were correctly diagnosed by fetal cardiac MRI; however fetal cardiac MRI missed 2 cases of associated pulmonary atresia (PA). Prenatal echocardiography (echo) correctly diagnosed five ASLBV and one retroesophageal LBCV as well as associated intracardiac anomalies. Fetal cardiac MRI can be a useful adjunct in the identification of subaortic and retroesophageal anomalous courses of the LBCV prenatally.

## Introduction

**Figure 1:**
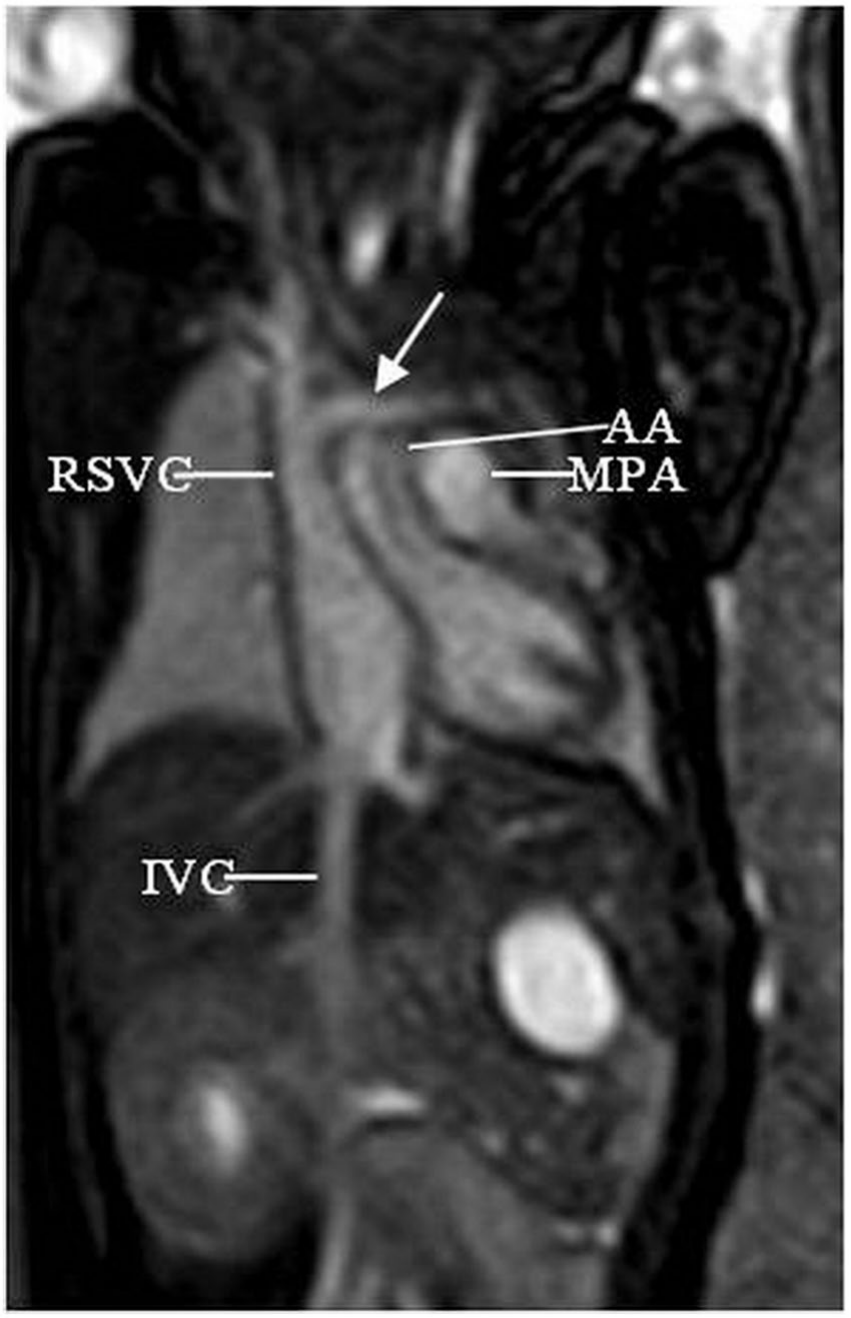
A 32-week fetus showing the normal brachicephalic course. Fetal cardiac magnetic resonance (CMR) steady-state free precession (SSFP) thoracic coronal view image shows the normal right sided superior vena cava (RSVC), left brachiocephalic vein (arrow), aortic arch (AA), main pulmonary artery (MPA), and inferior vena cava (IVC).

The left brachiocephalic vein (LBCV) is formed by the junction of the left subclavian and left jugular veins in the superior mediastinum. It receives venous return from the head, neck and left upper extremities. The normal course of the LBCV is obliquely downward, passing superior and anterior to the aortic arch and posterior to the thymus gland, then joining the right brachiocephalic vein to form the right sided superior vena cava (RSVC) (Fig. 1).

At times the LBCV can abnormally course below the aortic arch. This is known as a subaortic LBCV or a retroaortic LBCV^1,2^. Very rarely, the LBCV courses posterior to the esophagus, joining the azygos vein then draining into the superior vena cava. This is known as a retroesophageal or retrotracheal LBCV^3,4^. The descending portion of an anomalous LBCV should be differentiated from a persistent left superior vena cava (LSVC), ascending vertical vein in a total anomalous pulmonary venous connection and left partial anomalous pulmonary venous return^2^. The subaortic and retroesophageal anomalous courses of LBCV are often associated with additional congenital cardiovascular anomalies. Awareness of the presence of this is useful when planning transvenous pacemaker or central venous line placement^1,2^.

To the best of our knowledge only several ultrasound cases of the congenital anomalous course of LBCV have been reported worldwide with no cases diagnosed by fetal MRI^5–7^. In this study, we report ten cases of abnormal course of LBVC and describe the imaging technique and features by fetal cardiac MRI.

## Results

7282 fetal cardiac MRI were reviewed and 81 postnatal MRI/CT were reviewed for the presences of LBCV. 9 of 10 fetal cases with abnormal course of the LBCV were identified by fetal cardiac MRI and confirmed by postnatal MRI/CT, 1 of 10 fetal case with abnormal course of the LBCV was only identified by postnatal MRI during further imaging of the tetralogy of Fallot (TOF) with right aortic arch (RAA).

**Figure 2:**
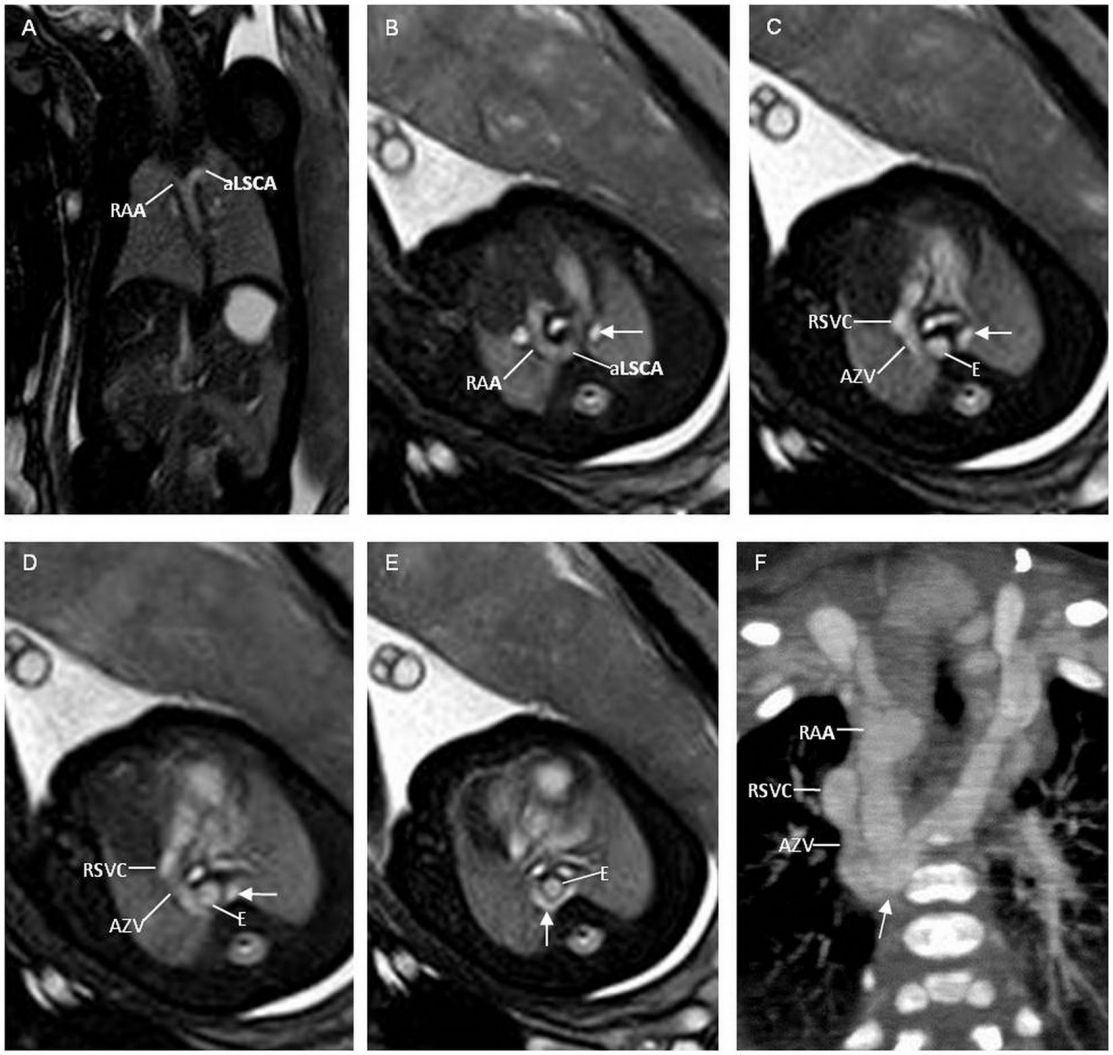
A 33-week fetus with a retroesophageal left brachiocephalic vein (LBCV) and a right aortic arch with aberrant left subclavian artery (RAA/aLSCA). Fetal CMR coronal view image of the aortic arch and sequential transverse view images show a right aortic arch (RAA in **A** and **B**) with an aberrant left subclavian artery (aLSCA in **A** and **B**), a LBCV (arrow in **B**), and a retroesophageal LBCV (arrows in **C**, **D**, and **E**) passing posterior to the esophagus (**E** in **C**, **D**, and **E**), joining the azygos vein (AZV in **C** and **D**), and finally draining into the superior vena cava (arrowheads in **C** and **D**); Postnatal CTA shows a right aortic arch (RAA in **F**) and a retroesophageal anomalous LBCV (arrow in **F**) joining the azygos vein (AZV in **F**) and finally draining into right superior vena cava (RSVC).

**Figure 3:**
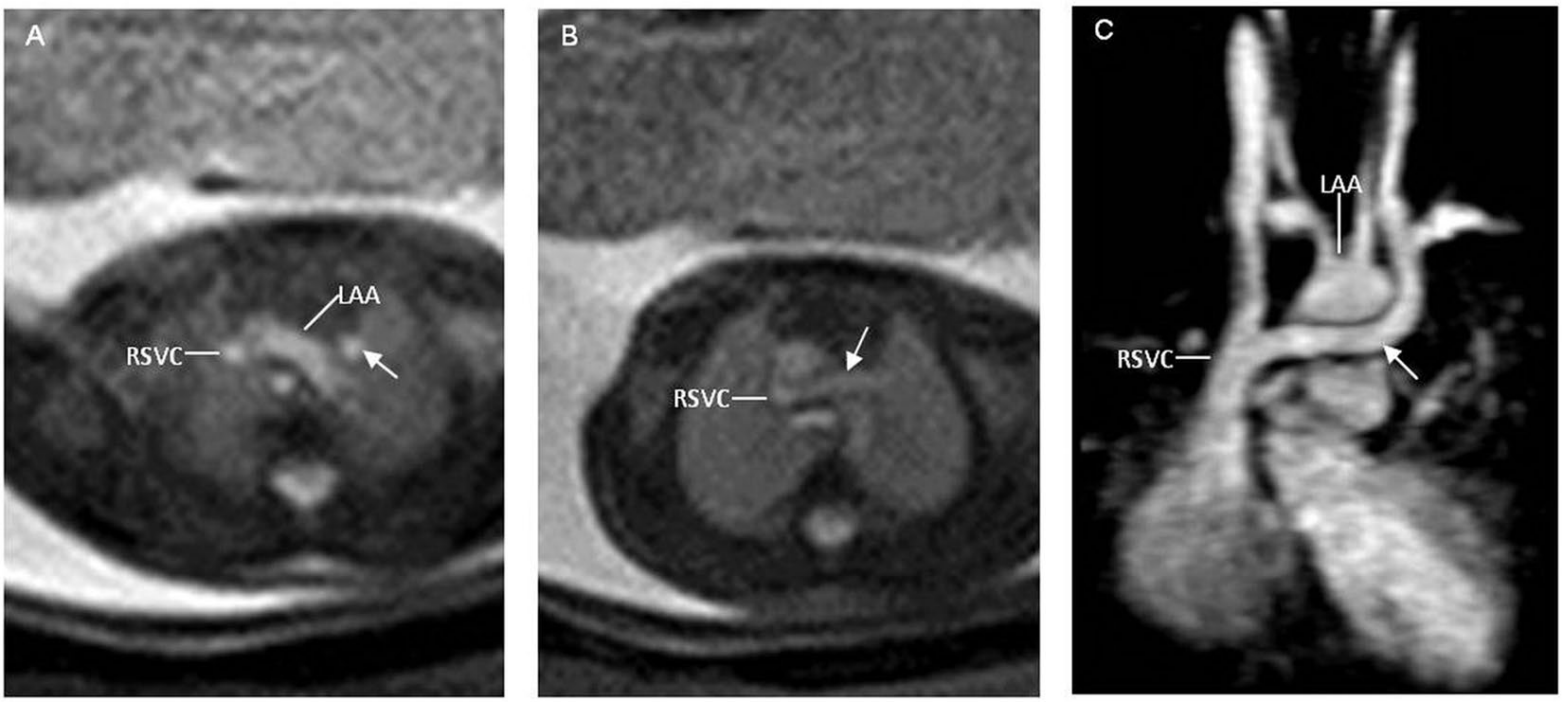
A 25-week fetus with an abnormal subaortic left brachiocephalic vein (ASLBV) with a left aortic arch. Fetal CMR B-TFE transverse view images show an abnormal subaortic left brachiocephalic vein (arrows in **A** and **B**) courses below the left aortic arch (LAA in **A**) into right superior vena cava (RSVC in **A** and **B**) and a left aortic arch (LAA in **A**); Postnatal MRI image also shows the left brachiocephalic vein (arrow in **C**) courses below the left aortic arch (LAA in **C**) into right superior vena cava (RSVC in **C**).

In 2 cases of retroesophageal LBCV identified by fetal MRI, the LBCV passed posterior to the esophagus, joining the azygos vein finally into the RSVC (Fig. 2). The results were confirmed by postnatal computed tomography angiography (CTA) in both cases (Fig. 2F). In 7 cases of sub-aortic LBCV, the LBCV abnormally coursed below the aortic arch, running between the ascending aorta and the trachea to reach the RSVC (Fig. 3A,B). The results were confirmed by postnatal MRI (Fig. 3C) or CTA.

**Figure 4:**
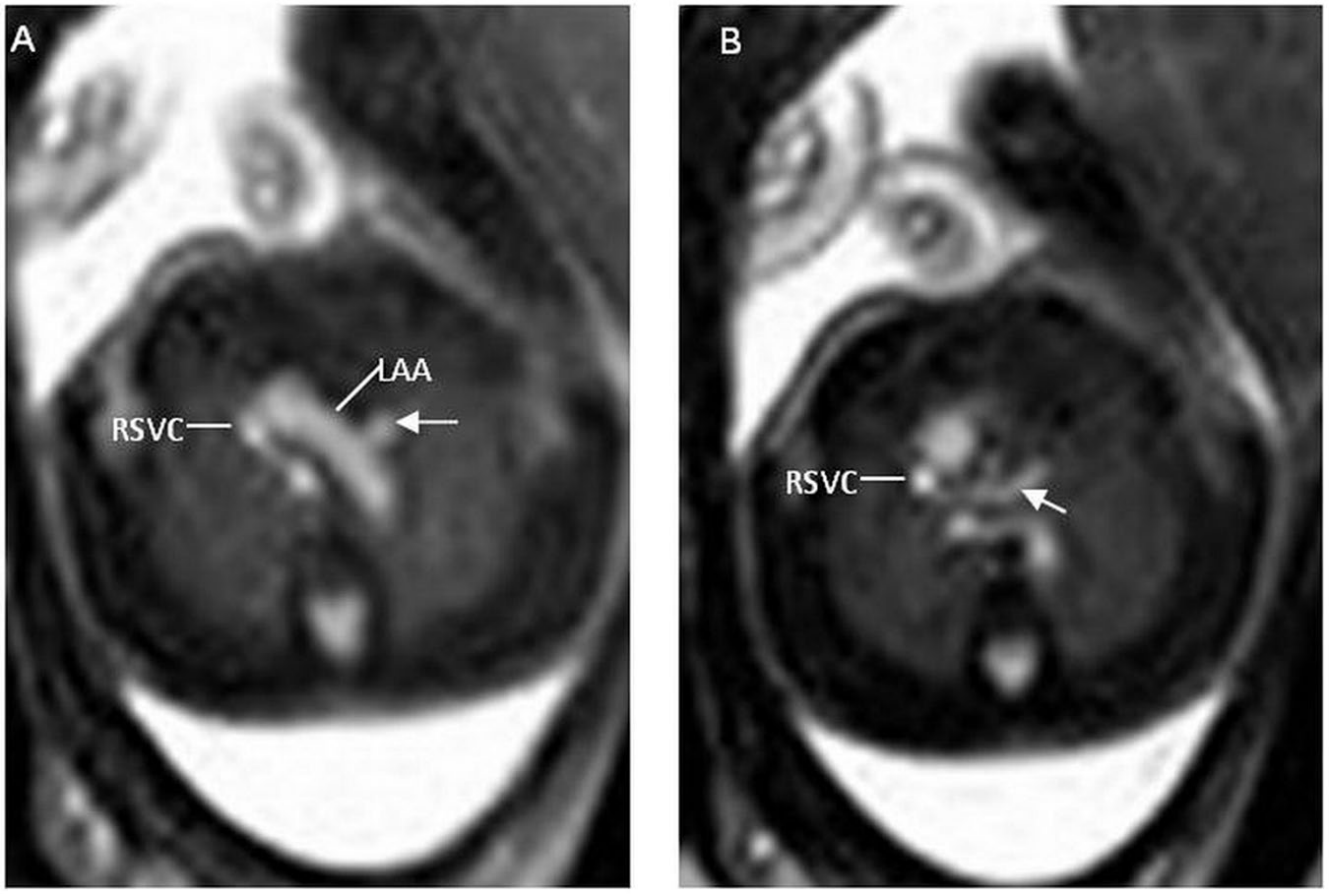
A 23-week fetus with an abnormal subaortic left brachiocephalic vein and pulmonary atresia with ventricular septal defect (PA/VSD). Fetal CMR B-TFE transverse view images show an abnormal subaortic left brachiocephalic vein courses (arrows in **A** and **B**) below the left aortic arch (LAA in **A**) into right superior vena cava (RSVC in **A** and **B**), transverse view image of the main pulmonary artery shows no main pulmonary artery.

5/10 cases had a right aortic arch (3 cases with right aortic arch with aberrant left subclavian artery (RAA/aLSCA),1 case with isolated RAA and 1 case with RAA had TOF). 5 cases had additional cardiac anomalies); two with pulmonary atresia and ventricular septal defect (PA/VSD) (Fig. 4), two with TOF and one with VSD.

Nine cases of anomalous courses of the LBCV were correctly diagnosed by fetal cardiac MRI, one case of ASLBV with RAA and TOF was missed by fetal MRI and identified postnatally during further imaging of the TOF. In 6 of 10 cases of anomalous courses of the LBCV, the diagnoses was also may by prenatal echocardiography (echo) including one case of retroesophageal LBCV and five cases of ASLBV (Table 1). Fetal cardiac MRI missed 2 cases of pulmonary atresia; Prenatal echo correctly diagnosed associated cardiovascular anomalies in all 10 cases.

**Table 1:** Demographics and comparison between prenatal echocardiographic results, fetal cardiac MRI and postnatal cardiac CT or MRI findings.

Cases	Maternal age (years)	GA at MRI (weeks)	Prenatal echo	Prenatal cardiac MRI	Postnatal enhanced cardiac CT/MRI
1	24	30	Retroesophageal LBCV, RAA/aLSCA	Retroesophageal LBCV, RAA/aLSCA	Retroesophageal LBCV, RAA/aLSCA
2	28	33	RAA/ aLSCA	Retroesophageal LBCV, RAA/aLSCA	Retroesophageal LBCV, RAA/aLSCA
3	22	25	ASLBV	ASLBV	ASLBV
4	33	29	ASLBV, RAA/aLSCA	ASLBV, RAA/aLSCA	ASLBV, RAA/aLSCA
5	28	23	ASLBV, RAA	ASLBV, RAA	ASLBV, RAA
6	35	23	ASLBV, PA/VSD	ASLBV, VSD	ASLBV, PA/VSD
7	29	24	PA/VSD	ASLBV, VSD	ASLBV, PA/VSD
8	22	25	RAA and TOF	RAA and TOF	ASLBV, RAA and TOF
9	24	25	ASLBV, TOF	ASLBV, TOF	ASLBV, TOF
10	32	26	VSD	ASLBV, VSD	ASLBV, VSD

LBCV: left brachiocephalic vein.

RAA/aLSCA: Right aortic arch with aberrant left subclavian artery.

ASLBV: abnormal subaortic left brachiocephalic vein.

RAA: Right aortic arch.

PA/VSD: pulmonary atresia and ventricular septal defect.

VSD: ventricular septal defect.

TOF: tetralogy of Fallot.

## Discussion

The brachiocephalic veins originate from the precardinal veins. At 8th embryonic week, the precardinal anastomosis between bilateral precardinal veins and develops into the LBCV^3^. The accessory hemiazygos veins develops from the left postcardinal vein. The left superior intercostal vein develops from the persistence of a small portion of the left common cardinal vein. The presence of a LSVC is the persistence of the proximal part of the left anterior cardinal vein. A retroesophageal LBCV mostly develops from the connection between the accessory hemiazygos vein and the left superior intercostal vein, with the interruption of the upper anastomosis, without the lower alternative anastomotic capillary plexus and persistent LSVC^3,4^. An ASLBV develops from the lower alternative anastomotic capillary plexus without persistent LSVC^1,2^.

Prenatal echocardiography is the primary tool to evaluate the fetal heart. The usual timing of fetal echocardiography for the prenatal diagnosis of congenital heart disease is approximately 20–24 weeks of gestation. During later gestation, reduction of amniotic fluid volume, increased ossification iof the ribs may decrease the image quality of fetal echocardiography. Fetal cardiac MRI at times can provide better imaging of the fetal heart. Some reports have described how fetal cardiac MRI can provide additional important information, particularly when obesity, uterine myoma, twin pregnancies and oligohydramnios are present^8,9^. With the technical developments of different novel gating approach for fetal cardiac MRI, fetal MRI can better both qualitative and quantitative evaluation of the fetal cardiovascular system^10–14^.

In this study, in 9 of 10 cases of anomalous LBCV, fetal cardiac MRI correctly diagnosed the anomalies with 1 case of retroesophageal LBCV and 3 cases of ASLBV missed by fetal echo; Prenatal echo however was important in the diagnosis of other associated cardiovascular anomaliesincluding pulmonary atresia.

The fetal cardiovascular MR imaging of the transverse view and the superior and inferior plane imaging of aortic arch are easy to obtain, and useful for interpretation. Transverse view of aortic arch is particularly important for visualization of the three vessels and trachea (3VT)^15^. Careful tracing of the vascular channels through sequential images is critical. ASLBV can be traced abnormally coursing below the aortic arch. The retroesophageal LBCV can be traced pass posterior to the esophagus, joining the azygos vein and finally draining into the superior vena cava. The persistent LSVC can be traced into an enlarged coronary sinus.

The subaortic or retroesophageal course of the LBCV is often associated with conotruncal and aortic arch anomalies and can be associated with genetic anomalies including 22q11 deletion^1,5,6^. The identification of LBCV prenatally suggests the need for genetic testing. For this series, genetic testing however was not available.

Prenatal identification of LBCV presents a clue to search for additional cardiac anomalies and aneuploidy. Postnatally, awareness of LBCV can guide central line and pacemaker insertion planning.

Several limitations of this series are worth noting. This was a retrospective study and as such was subject to limitations associated with its study design. The MRI readers were not blinded to the diagnosis by fetal echo. This study did not include the evaluation of fetal intrathymic anomalous courses of the LBCV and no genetic testing was offered.

In summary, fetal cardiac MRI can be a useful adjunt in the evaluation of subaortic and retroesophageal anomalous courses of the left brachiocephalic vein. Its identification warrants a closer look for intracardiac anomalies.

## Methods

The ethics commission of Shanghai Children’s Medical Center approved this fetal MRI study. All pregnant mothers involved in the study provided written informed consent for use of their clinical data for research purposes prior to the examination. All methods of the study were performed in accordance with the relevant guidelines and regulations.

### Study subjects

From June 2006 to March 2017, 7282 fetal MRI’s were performed. Using review software, we reviewed both databases of fetal MRI and postnatal cardiac CT/MRI, Ten fetal MRI cases were confirmed postnatally to have anomalous courses of LBCV by cardiac CTA or MRI. The gestational age of these fetuses at time of fetal MRI ranged from 23 to 33 weeks (mean, 27 weeks). The age of the pregnant women ranged from 21 to 40 years (mean 30 years). Fetal cardiac MR examination was performed usually within 2 days after a detailed fetal echo which resulted in a request for the fetal MRI.

### Magnetic resonance imaging

Fetal MRI was performed using a 1.5 T unit (Signa Echospeed; GE Medical Systems, Milwaukee, WI and Achieva Nova dual; Philips Medical Systems, Best, The Netherlands). Imaging sequences included a multiplanar steady-state free-precession (SSFP) sequence, non-gated cine SSFP sequence (TR/TE, 2.7/1.34 ms; field of view, 280–310 mm^2^; section thickness, 8–10 mm; spacing, −2 to −4 mm; flip angle, 50°; 20 images obtained, repeated 2 times; total scan time, 23 second) and single-shot turbo spin echo (SSTSE) sequence. SSFP sequences (TR/TE, 3.6/1.8 msec; field of view, 260–325 mm^2^; section thickness, 4–6 mm; overlapping, 3–5 mm; matrix, 192 × 193–216 × 218; flip angle, 70–80°) were acquired along the transverse, four-chamber, short-axis, coronal and oblique sagittal views of the fetal thorax and heart, SSTSE sequences (TR/TE, 12000/120 msec; field of view, 280–335 mm^2^; section thickness, 46 mm; spacing, 0knes mm; matrix, 192 × 193–236 × 220; flip angle, 90, were acquired in order to show the bronchus and for evaluation of the visceroatrial situs, especially transverse views of the fetal thorax. No sedatives, no intravenous gadolinium-based contrast media and no fetal cardiac gating were used.

Postnatal cardiac CTA or MRI were performed between the ages of 1–12 months to confirm the prenatal diagnosis and evaluate the airway and associated cardiovascular anomalies. The diagnosis of anomalous courses of LBCV was confirmed by postnatal imaging (cardiac CTA or MRI) in all cases.

## Data Availability

The datasets generated and analyzed during the current study are available from the corresponding author on reasonable request.
